# Fetal mri of obstructive hydrocephalus: a review proposing a surgical etiology-based approach

**DOI:** 10.1007/s00234-025-03827-3

**Published:** 2025-11-21

**Authors:** Mario Tortora, Francesco Pacchiano, Chiara Doneda, Filippo Arrigoni, Giana Izzo, Ferdinando Caranci, Fabio Tortora, Cecilia Parazzini, Kshitij Mankad, Andrea Righini

**Affiliations:** 1 Neuroradiology Unit, Department of Advanced Biomedical Sciences, Federico II University, Naples, Italy; 2 Pediatric Radiology and Neuroradiology Unit, V. Buzzi Childrens Hospital, Milan, Italy; 3 Department of Precision Medicine, University of Campania “L. Vanvitelli”, Caserta, Italy; 4 Neuroradiology department, Great Ormond Street Hospital, London, UK

**Keywords:** Ventriculomegaly, Hydrocephalus, Fetal MRI, CSF

## Abstract

**Introduction:**

Ventriculomegaly (VM) is the fetal central nervous system (CNS) anomaly most commonly represented in prenatal imaging. It is defined as a lateral ventricle of dimensions greater than or equal to 10 mm; it can be unilateral or bilateral. More generally, hydrocephalus is defined as an imbalance between brain parenchyma and cerebrospinal fluid (CSF) due to an abnormal increase of the latter within the ventricles in an almost bilateral manner. To identify ventriculomegaly and categorize its severity, the appropriate imaging and measurement methods are crucial. Clinical outcomes vary greatly because of the wide differential diagnosis. Furthermore, there is a significant chance that these causes may recur in subsequent pregnancies. Pregnancy care and counseling depend on a precise diagnosis of the underlying cause.

**Materials and methods:**

We retrospectively reviewed our institutional fetal MR imaging database (4568 examinations) from 2005 until 2024. We focused on obstructive hydrocephalus and, according to rigorous inclusion/exclusion criteria (Table 1), we enrolled 201 cases.

**Results:**

We analyzed isolated aqueduct stenosis (36.3%); hemorrhagic events (30.3%); rhombencephalosynapsis (7.5%); dural sinus malformation (6%); midline cysts (5.4%); diencephalic-mesencephalic junction (DMJ) dysplasia (3.5%); infectious lesions (3%); tumors (2.5%); Chiari 1 (1.5%); Walker Warburg disease (1%); not otherwise specified (3%).

**Discussion:**

We discuss the different etiologies of obstructive hydrocephalus in our population and propose an etiology-based approach that allows the clinician and radiologist to reach the correct differential diagnosis and provide an indication for possible fetal surgery.

**Conclusion:**

Hydrocephalus arises from embryological abnormalities or acquired insults, requiring precise neuroimaging for diagnosis and management. A thorough imaging approach aids in etiological diagnosis, surgical planning, and essential counseling.

## Introduction

**Fig. 1:**
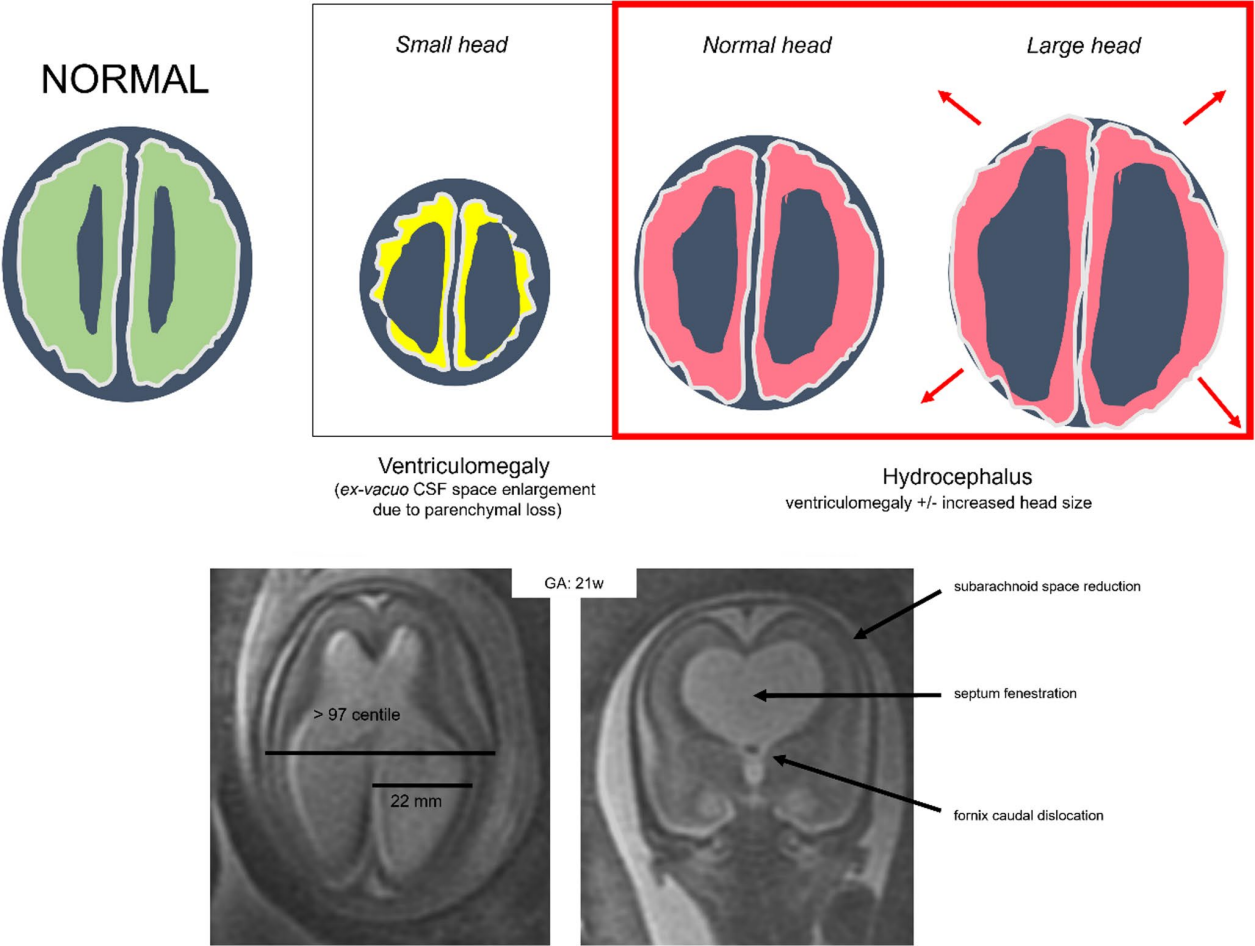
Illustrations show, compared with a normal case, our practical definition of fetal obstructive hydrocephalus and its cardinal features: abnormal increase of global intracranial CSF with ventricular size >10 mm (22mm, in this case at 21-week GA) and skull biparietal diameter >97 centile; subarachnoid space reduction; septum fenestration and fornix caudal dislocation

Hippocrates was the first to identify hydrocephalus, one of the oldest neurological conditions known. The Greek words ὑδρo (hydro) and κεϕαλή (cephalous), which translate to “water on the brain”, represent the term’s etymologic roots [1]. According to our practical definition, obstructive fetal hydrocephalus refers to global intracranial cerebrospinal fluid (CSF) volume increase with respect to normal, once microcephaly and ex-vacuo ventriculomegaly (VM) have been excluded. In this regard, it is mandatory to search for: enlarged ventricles more than 10 mm; reduction of peri cerebral spaces; septum fenestration and caudal dislocation of the fornix, as shown in Fig. 1.

**Fig. 2:**
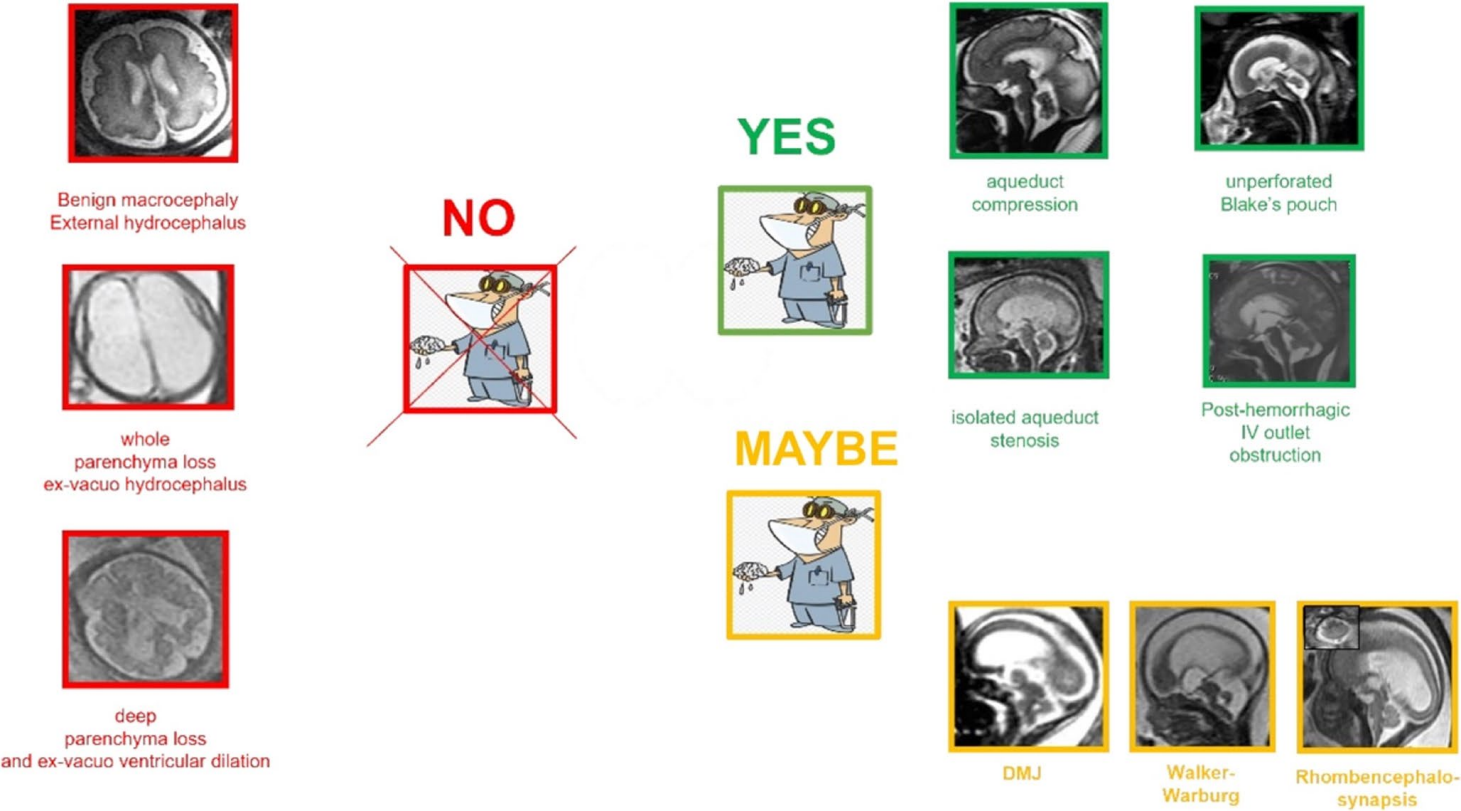
**Pre-surgical evaluation**: decision-making clues, based on our experience, that might guide the radiologist and gynecologist to hypothetical neurosurgical planning in the future. In detail, we identify three scenarios in which intrauterine shunting surgery is: theoretically likely (green); very doubtful (yellow); very likely not indicated (red)

Even though hydrocephalus can afflict anyone at any age, pediatric hydrocephalus affects 1 in 1,000 live births and is the most frequent cause of brain surgery in young patients [2]. Additionally, it is the most commonly depicted abnormality of the fetal central nervous system (CNS) at prenatal imaging. Although this finding is nonspecific, it has been recognized as a marker of aberrant fetal central nervous system (CNS) development with a rate of associated malformations ranging from 10% to 50% [3–5]. The incidence of associated anomalies increases with the degree of ventriculomegaly [6]. MRI plays a pivotal, complementary role to US in characterizing ventriculomegaly/hydrocephalus and in detecting associated anomalies relevant to counseling and management [7, 8]. Literature shows that ventricular dilatation was considered as isolated on US in 85% of cases, whereas this percentage drops to 45% of cases following MRI examination [9]. The prognosis varies based on the underlying illness. In this regard, we believe it is necessary to stress the concept according to which each ventriculomegaly should be examined in depth with an MRI examination. It is necessary to make a correct etiological diagnosis and provide correct diagnostic-prognostic counseling that also includes the possibility of resorting to surgical intervention in utero. This article proposes a surgical approach based on etiology as described in Fig. 2.

This article provides examples of the most common causes of VM observed at MR after a thorough retrospective revision of our institutional database. The focus is on suggestions for differential diagnosis, prognosis, risk of recurrence, and therapeutic indications.

Our study could serve as a reference point for the appropriate management of in utero therapy for obstructive hydrocephalus. Historical guidelines for fetal surgery, established in 1981, are currently under revision following a moratorium. The poor outcomes reported at that time were largely related to the absence of fetal MRI, which today allows accurate case selection and characterization. Therefore, those early recommendations are no longer applicable in the current era.

It is also important to clarify that the neurosurgical techniques described in the literature primarily refer to postnatal management. Nevertheless, potential adaptations of similar approaches could, in the future, be investigated at the fetal stage. To date, the only experimental experience available concerns galenic aneurysmatic cyst, while there are no recent reports in the literature of fetal drainage procedures for other etiologies of obstructive hydrocephalus. This underscores both the novelty and the potential translational impact of our findings, as they open the way to rethinking therapeutic strategies for carefully selected cases.

### Anatomy and embryology of the ventricular system

**Fig. 3:**
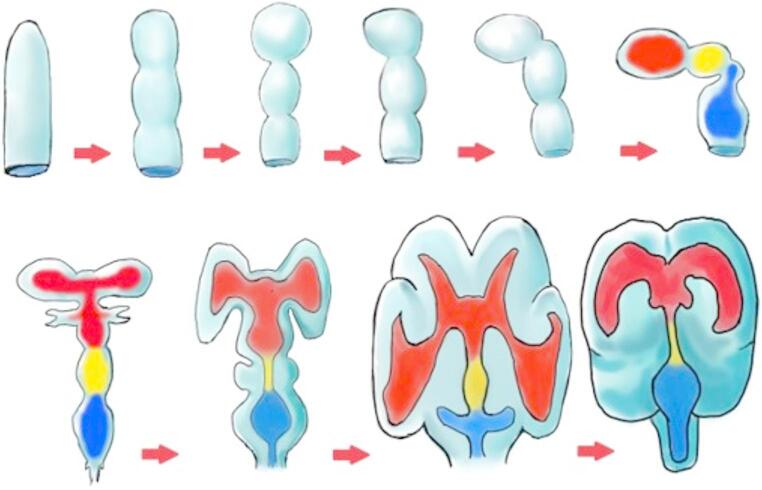
Illustration shows stages of ventricular system embryogenesis

Embryologically, following neural tube closure, three dilatations, the primitive brain vesicles, are formed on the cephalic end of the neural tube. These vesicles, from cephalic to caudal, are the prosencephalon (forebrain), mesencephalon (midbrain), and rhombencephalon (hindbrain). At the fifth week of gestation, the prosencephalon gives rise to the telencephalon (cerebral hemispheres) and the diencephalon. The rhombencephalon also gives rise to the metencephalon (cerebellum and pons) and the myelencephalon (medulla oblongata). Cavities remain inside these developing vesicles, communicating with the lumen of the neural tube and represent the future ventricles. The cavity of the rhombencephalon is the fourth ventricle, the cavity of the diencephalon is the third ventricle, and those of the telencephalon are the lateral ventricles. The lumen of the mesencephalon connects the third and fourth ventricles and later becomes narrower and known as the cerebral aqueduct [22]. During the early phase of their development, the ventricles undergo massive expansion with faster growth compared to the surrounding brain tissue. Upon reaching the maximum ventricle/brain ratio, with the ventricle assuming the adult size, the brain begins to outpace the ventricular growth and leads to a change of the ventricular configuration to the adult form. This process proceeds in a caudal to rostral direction [23] (Fig. 3).

**Fig. 4:**
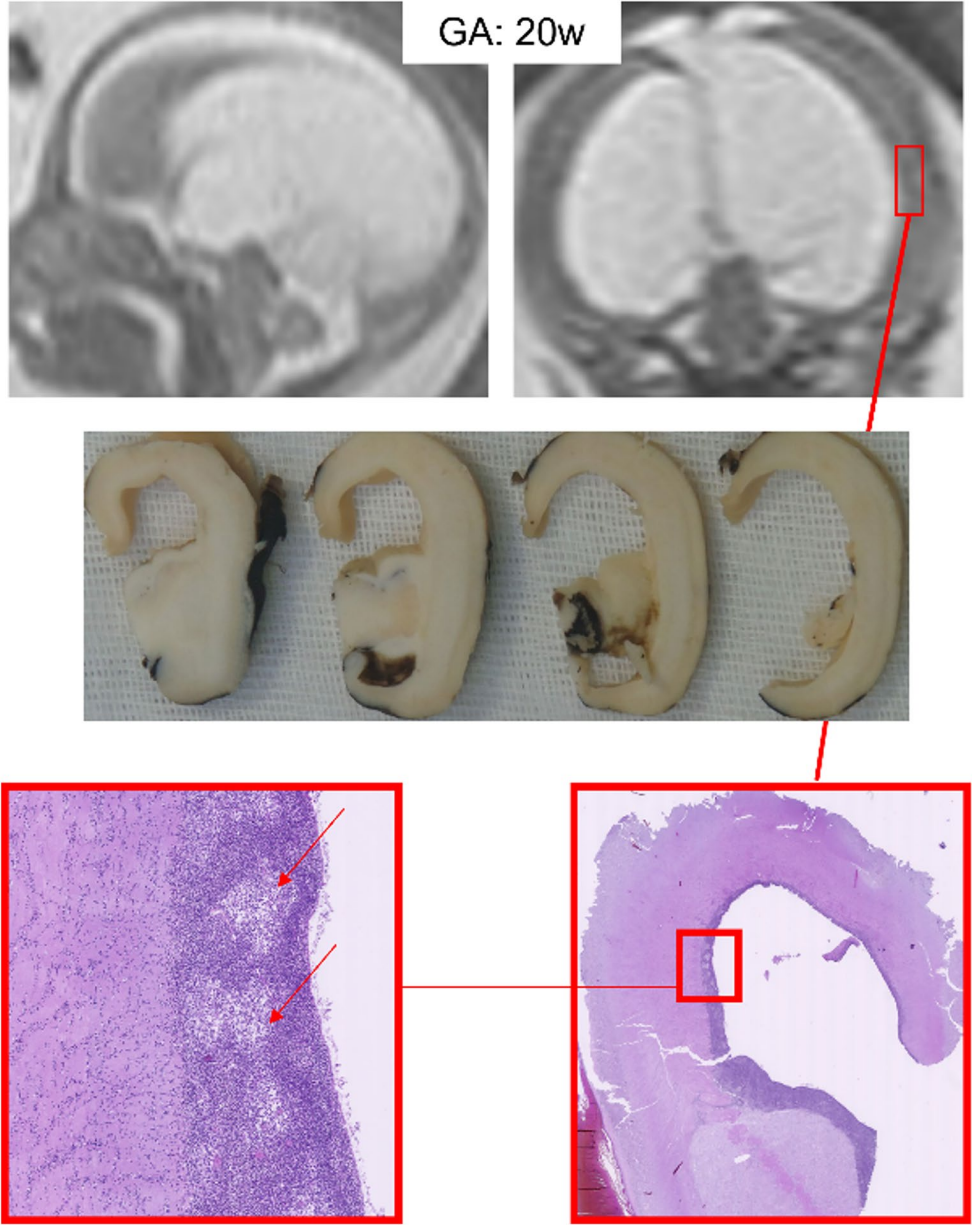
**Detrimental effect on normal cell proliferation and migration**: sagittal and coronal single-shot fast spin-echo T2-weighted MR images of a 20-week fetus confirming US suspicious of obstructive hydrocephalus; the couple decided to terminate the pregnancy. At autopsy, multiple gross brain sections were obtained. Optical microscopy analysis revealed (arrows and boxes) a rarefaction of areas typically involved in active cell proliferation, partially replaced by foci of necrosis, suggesting a detrimental effect on the normal processes of neuronal proliferation and migration. This alteration indicates a possible impairment of corticogenesis, with potential implications for cortical layer formation and the architectural organization of the brain tissue

The ventricular system of the brain consists of four cavities, including the two lateral ventricles, the third ventricle, and the fourth ventricle. These cavities are believed to be related to the development of the frontal, parietal, and occipital lobes, which displace the temporal lobe. Each lateral ventricle is divided into a body, atrium, and anterior, posterior, and inferior horns. The body occupies the parietal lobe and extends from the posterior edge of the foramen of Monro to the point where the septum pellucidum disappears and the corpus callosum and fornix meet [10–12]. Local ventricular volume is heritable and increases with age from childhood to late middle age. Asymmetry of the lateral ventricles exists, with an incidence of 5–12% [13–15]. The degree and character of right-to-left asymmetry also changes with various diseases and abnormalities. For example, in patients with autism, highly localized reductions in volume in the left frontal and occipital horns of the lateral ventricles have been reported [16]. The interventricular foramen, a communicating canal between the lateral ventricle on either side and the third ventricle, has a diameter of 3–4 mm and is bounded anteriorly by the junction of the body and the columns of the fornix and posteriorly by the anterior pole of the thalamus. Congenital atresia of the foramen of Monro is reported [17, 18]. The third ventricle is a narrow, funnel-shaped, midline cavity at the center of the head, connected to the lateral ventricles through the interventricular foramen of Monro and the cerebral aqueduct of Sylvius [17]. The aqueduct of Sylvius is the narrowest part of the ventricular system and the most common site for blockade. The fourth ventricle is a broad, tent-shaped midline cavity located at the center of the posterior fossa between the brain stem and the cerebellum. It is connected rostrally with the cerebral aqueduct, caudally with the central canal of the spinal cord, infero-posteriorly with the cisterna magna through the foramen of Magendie, and laterally with the cerebellopontine angles through the foramena of Luschka [18–20]. Ependyma, a type of cuboidal to columnar epithelial cells, line the ventricular system of the brain and central canal of the spinal cord. In the third ventricle, tanycytes, unique elongated cytoplasmic processes, extend from the ventricular lining toward the surrounding neuropils, enwrapping blood vessels or terminating on neurons, glia, or the external glial limitans. This structure suggests a role in neuroendocrine connection between the CSF and the hypophysial-portal vasculature, affecting the adenohypophysis function along with the hypothalamus [20]– [21]. Obstructive hydrocephalus in the fetus and newborn is considered potentially capable of altering normal brain development, having as its *primum movens* ependymal damage. This inflicts a harmful effect on normal cell proliferation and migration, which can be detected under the optical microscope through the rarefaction of areas of active cell proliferation (Fig. 4). In fact, our hypothesis is that ependymal damage secondary to obstructive hydrocephalus leads to increased neuronal apoptosis, hypoxic damage and dysfunction in the proliferation and differentiation processes of neural stem cells resulting in inflammation or scar tissue formation. Such substrates are also responsible for secondary cortical malformations and myelination disorders.

The brain ventricles’ precise position and shape are controlled by various patterning genes and their products, such as Sonic Hedgehog (*SHH*), *H1 × 1*, and *ZIC* family [24]. Early separation from the notochord may lead to loss of Shh expression and ventricular collapse. The shape of the ventricles is a result of uneven cellular proliferation, migration, and differentiation throughout the neural tube, controlled by patterning genes in different brain regions. Programmed cell death also plays a role in controlling proper ventricular formation. In addition to spatial morphogenesis, specific tissue changes are genetically regulated, including cell shape changes, cytoskeleton formation, intercellular adhesion, tissue maintenance, and epithelial anchoring. The embryonic CSF, secreted by neuroepithelial and non-neuroepithelial sources, plays a crucial role in ventricle development by creating intraluminal pressure and containing proteins and growth factors [23]. Studies of familial congenital hydrocephalus have identified several genes that cause disease with X-linked (*L1CAM* and *AP1S2*) or autosomal recessive (*MPDZ* and *CCDC88C*) inheritance [25–27]. More recent studies using whole exome sequencing have found that damaging de novo gene mutations account for about 20% of patients with sporadic (non-familial) congenital hydrocephalus, with *TRIM71*, *PTEN*, *SMARCC1*, *FOXJ1*, and *PIK3CA* exhibiting exome-wide significant enrichment of de novo mutations [28–30]. In addition, over 100 other genes have been described to be mutated in syndromic hydrocephalus [31]. Although hydrocephalus-associated genes have diverse molecular functions and are involved in numerous pathways, integrative genomic analyses have revealed the convergence of these genes in cortical neurogenesis elements [32], contrary to the dogma that hydrocephalus arises from abnormal fluid hydrodynamics. Beyond *MPDZ* and *L1CAM*, involved in adhesion/junction functions [33, 34], *CRB2-* cell polarity promoter [31, 35] and *DNAAF5*, a dynein motor assembly factor, are associated with the autosomal heterogenic recessive condition of hydrocephalus [36].

## Materials and methods

This is a retrospective monocentric (Children’s hospital of Milano, “Vittore Buzzi”); we retrospectively reviewed our institutional fetal MR imaging database (4568 exams) since 2005 until 2024. Focused on obstructive hydrocephalus, according to rigorous inclusion/exclusion criteria (Table 1), we enrolled 201 cases. The selection criteria are described in detail in the results as well as the demographic data. The candidates were recruited as clinical cases with ethical approval for retrospective review of clinical notes and MRI by the Milano Area 1 Ethics Committee (n. 27757/2022). Informed consent to the research and to publication of the results was obtained.

Intrauterine MRI (iuMRI) was performed on a 1.5 Tesla scanner (Achieva, Philips Medical Systems) using a phased array abdominal coil, with a protocol that included multiplanar single-shot T2-weighted fast spin-echo (FSE) slices (TR/TE, 3000/180 ms; voxel size, 1.25 × 1.98 × 3 mm; gap, 0.1 mm; in-plane resolution, 1.02 mm2), multiplanar balanced steady-state (B-TFE) slices (TR/TE, 7.3/3.7 ms; voxel size 1.47 × 1.46 × 2 mm; gap 0; in-plane resolution 1.05 mm2), multiplanar T1-weighted FSE slices (TR/TE, 300/14 ms; turbo factor, 3; in-plane resolution, 1.4 mm2) and, in some cases, axial DWI slices (TR/TE, 1000/90 ms; b-factor, 0–600 s/mm2). All included studies had imaging quality that we deemed sufficient, and the included scans met the minimum requirements of the ISUOG guidelines for fetal MRI.

Postmortem/postnatal (pmMRI) was performed on both 1.5 and 3.0 Tesla scanners On 1.5 Tesla scanner, we used the smallest coil that fit the fetus, with a protocol including high-resolution multiplanar T2-weighted FSE sections (TR/TE, 6000/200 ms; voxel size, 0.3 × 0.3 × 2 mm; in-plane resolution 0.26 mm2; NEX 7), multiplanar T1-weighted SE sections (TR/TE, 406/12 ms; voxel size 0.45 × 0.45 × 2 mm; NEX 5), and sometimes multiplanar BALANCE B-TFE sections (TR/TE, 7/3.5 ms; voxel size 0.59 × 0.59 × 1 mm). On 3 Tesla scanner, the protocol consisted of high-resolution multiplanar T2-weighted FSE sections (TR/TE, 6500/120 ms; voxel size 0.3 × 0.3 × 1.2 mm; NEX 4), sagittal tridimensional T1-weighted fast-field echo sections (TR/TE, 8/4.6 ms; voxel size 0.7 × 0.7 × 0.7 mm; NEX 4).

Fetal brain tissue samples were obtained from human fetuses following legally authorized elective pregnancy terminations. The collection and use of the samples were conducted in accordance with current regulations and approved by the Ethics Committee, with written informed consent obtained from all donors. Brain tissues were fixed in 10% neutral-buffered formalin, processed using standard paraffin-embedding protocols, and sectioned at 5 μm for histological analysis using conventional staining methods.

We performed only descriptive statistics, including frequencies and percentages.

**Table 1:** inclusion/exclusion criteria

Inclusion Creteria	Exclusion Criteria
Ventriculomegaly	Callosal agenesis
Thinned brain mantle	Chiari 2 **with** cephaloceles
Cephalic measurements ruling out microcephaly	Holoprosencephaly or Hydranencephaly
Effaced pericerebral spaces with or without ruptured septum or cortical rim dehiscence	Ventriculomegaly associated with CSF pericerebral space enlargement
Site of obstruction identified or suspected	Mild unilateral ventriculomegaly

## Results

We found 2,457 cases with ventriculomegaly that satisfied the inclusion criteria out of the 4,568 examinations. Of them, 1,621 (65.9%) examples of bilateral ventriculomegaly were chosen after excluding 836 unilateral cases. 841 (51.9%) of these were excluded because of agenesis of the corpus callosum, Chiari 2 with cephalocele, or holoprosencephaly/hydranenecephaly. For a final sample of 201 recruited cases, 579 examinations from the remaining 780 instances were disqualified because they either did not have a definitive obstructive etiology or were an indication of ventriculomegaly linked to CSF pericerebral space enlargement. 72% of the MR scans were performed between 18 and 21 weeks of gestation.

Finally, we analyzed isolated aqueduct stenosis (73 cases; 36.3%); hemorrhagic events (61 cases; 30.3%); rhombencephalosynapsis (15 cases; 7.5%); dural sinus malformation (12 cases; 6%); midline cysts (11 cases; 5.4%); diencephalic-mesencephalic junction (DMJ) dysplasia (7 cases; 3.5%); infectious lesions (6 cases; 3%); tumors (5 cases; 2.5%); Chiari 1 (3; 1.5%); Walker Warburg disease (2 cases; 1%); not otherwise specified (6 cases; 3%).

## Discussion

### Isolated aqueductal stenosis

**Fig. 5:**
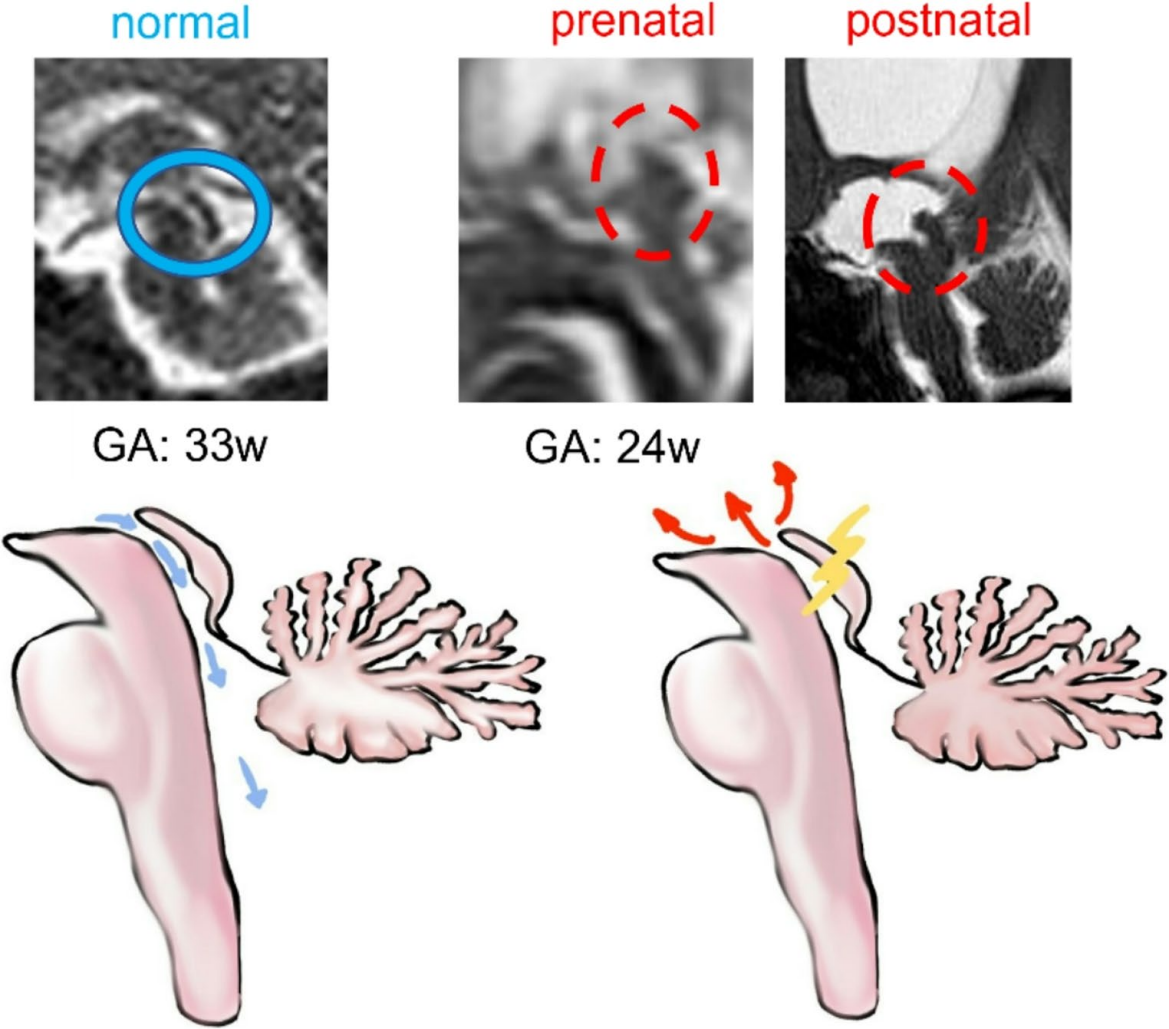
A normal fetus (33w) compared with a case of hydrocephalus due to isolated aqueductal stenosis (MRI at 24w and at birth). Illustrations show how obstruction causes a reversal of the CSF flow and an accumulation in the supratentorial areas

The most common cause of severe ventriculomegaly is aqueductal stenosis, which results from narrowing of the cerebral aqueduct of Sylvius located between the third and fourth ventricle leading to progressive dilatation of the lateral and third ventricles (Figure 5).

**Fig. 6:**
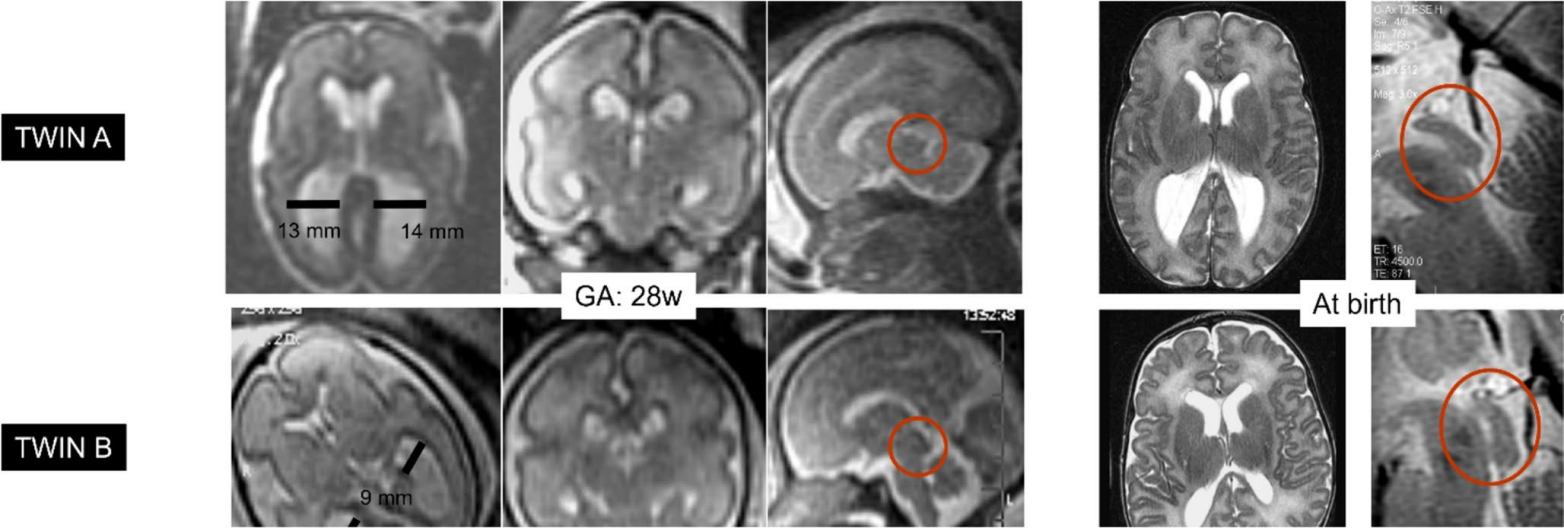
Monochorionic twins: an interesting paradigm of genetic-based aqueductal stenosis (autosomic recessive). Prenatal and postnatal T2-weighted images show stenosis of the lower third of the aqueduct, more evident in twin A, affected by moderate ventriculomegaly

The overall incidence of aqueductal stenosis is 0.5 to 1 per 1,000 births [37]. Aqueductal stenosis (AS) can be genetic (Fig. 6) or can result from fibrosis secondary to fetal infection (e.g., cytomegalovirus [CMV], toxoplasmosis, or Zika virus) or bleeding as intraventricular hemorrhage.

**Fig. 7:**
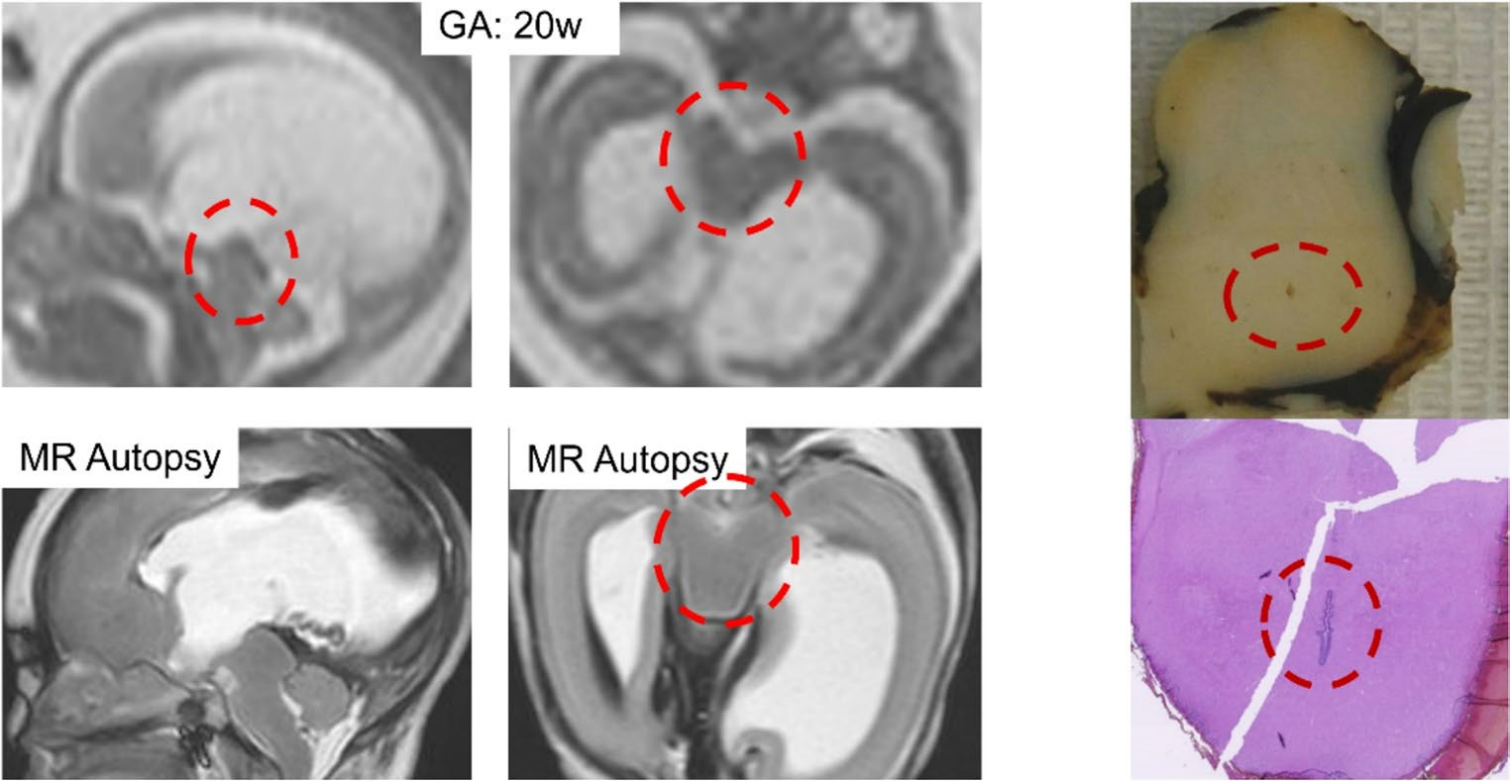
Sagittal and axial single-shot fast spin-echo T2-weighted MR images of a 20-week fetus and corresponding MR autopsy sections confirming stenosis of Sylvian aqueduct. Gross and microscopic anatomy makes the anomaly even more evident as the aqueduct appears only barely visible

A mass or congenital tumor can also lead to compression of the aqueduct with resultant ventriculomegaly [38]. Hydrocephalus related to CRB2, MPDZ and CCDC88C constitutes a separate pathogenic group of congenital hydrocephalus with atresia of Sylvius aqueduct [39]. In 5% of all cases, an X-linked transmission, known as Bickers–Adams syndrome may be the etiology. This genetic disorder, which is also known as X-linked hydrocephalus and hereditary stenosis of the aqueduct of Sylvius (HSAS), is related to mutation of the *L1CAM* gene on chromosome Xq28 [33]. In many cases, the cause of aqueductal stenosis may remain unknown. Increased access to whole exome sequences (WES) research could identify new causes of aqueduct stenosis [40]. Others suggest that the aqueduct may inherently become narrowed, particularly at the superior colliculus or intercollicular sulcus. In some cases, the aqueduct can be “forked,” with branching dorsal and ventral channels. Less commonly, a web can develop along the most inferior recess of the aqueduct [41]. On fetal MRI, the aqueduct of Sylvius is well depicted already from 20-week (Fig. 7).

With obstruction to CSF flow, there can be complete or partial obliteration of the CSF space in the aqueduct [42]. The tectum may be deformed or thickened. The lateral and third ventricles are dilated. Ventricular disruption is sometimes depicted in the posterior mesial aspects of the brain, the location of the choroidal fissure and weakest part of the ventricular wall [40]. This results in a focal ventricular diverticulum and thinning of the adjacent brain parenchymal mantle. Most disruptions are lined with white matter and are thus not consistent with schizencephaly. The septum pellucidum can be partially or completely absent. Although MRI can visualize the corpus callosum, in the presence of severe VM, it is often displaced, severely stretched. The subarachnoid spaces may be normal or absent depending on gestational age, and sulcation is typically delayed. The posterior fossa may be small owing to mass effect. Outcome in fetuses with AS is guarded. Overall mortality is 40%, perinatal mortality of 23% and of those that survive only 10% have normal development [43]. Prognosis has been shown to depend on age at diagnosis as progressive increased intracranial pressure can result in further insult to the brain; presence of additional anomalies, karyotype abnormality, and in utero progression result in a negative outcome [44]. In the presence of AS or severe ventriculomegaly, termination may be considered. Most fetuses with enlarged cranium require delivery by C-section. Postnatally, most infants require ventricular shunting.

### Hemorrhagic forms

**Fig. 8:**
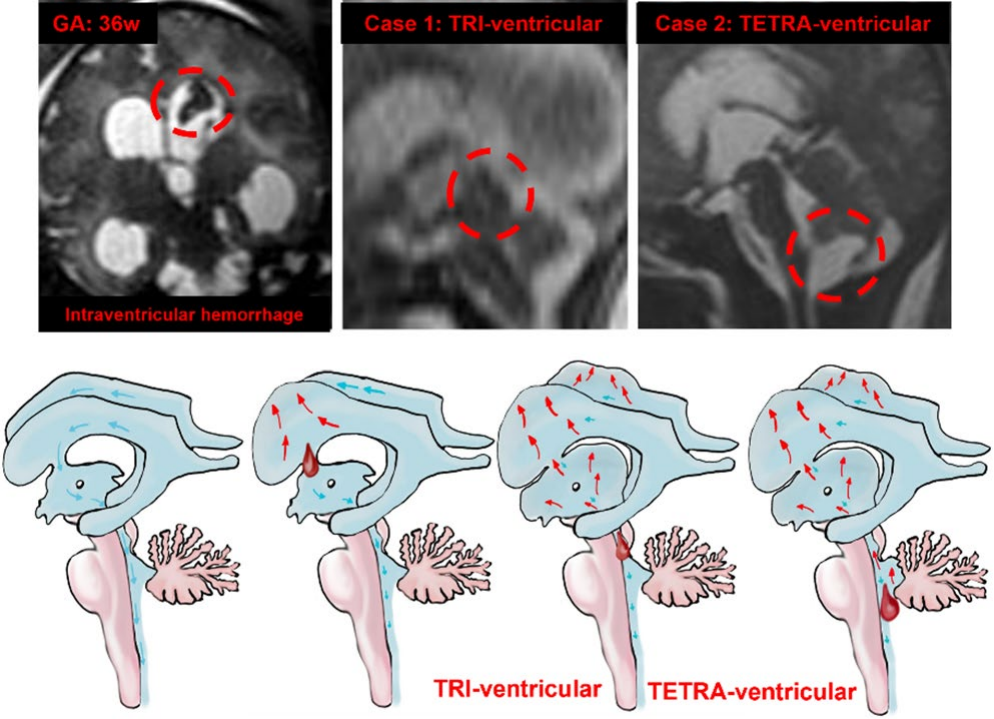
Illustrations show how, based on the location of the obstruction, a reversal of CSF flow and an accumulation is created which can affect the supratentorial (tri-ventricular) and infratentorial (tetra-ventricular) sections

Post-hemorrhagic hydrocephalus commonly occurs due to premature birth, a condition in which the highly vascularized and fragile germinal matrix (an area of periventricular brain parenchyma) is prone to bleed under stress, allowing entry of blood into the adjacent ventricular system. A CSF hydrodynamic mechanism is classically invoked to explain the pathogenesis of post-hemorrhagic hydrocephalus: obstructive ventriculomegaly occurs due to physical obstruction of intraventricular CSF flow by blood micro-thrombi with subsequent inflammation and fibrosis of the arachnoid granulations leading to reduced CSF clearance from the ventricular system [45]. Denudation of the multiciliated ependyma following intraventricular hemorrhage contributes to the pathological accumulation of CSF secondary to impaired cilia-driven CSF circulation [46, 47] (Fig. 8).

**Fig. 9:**
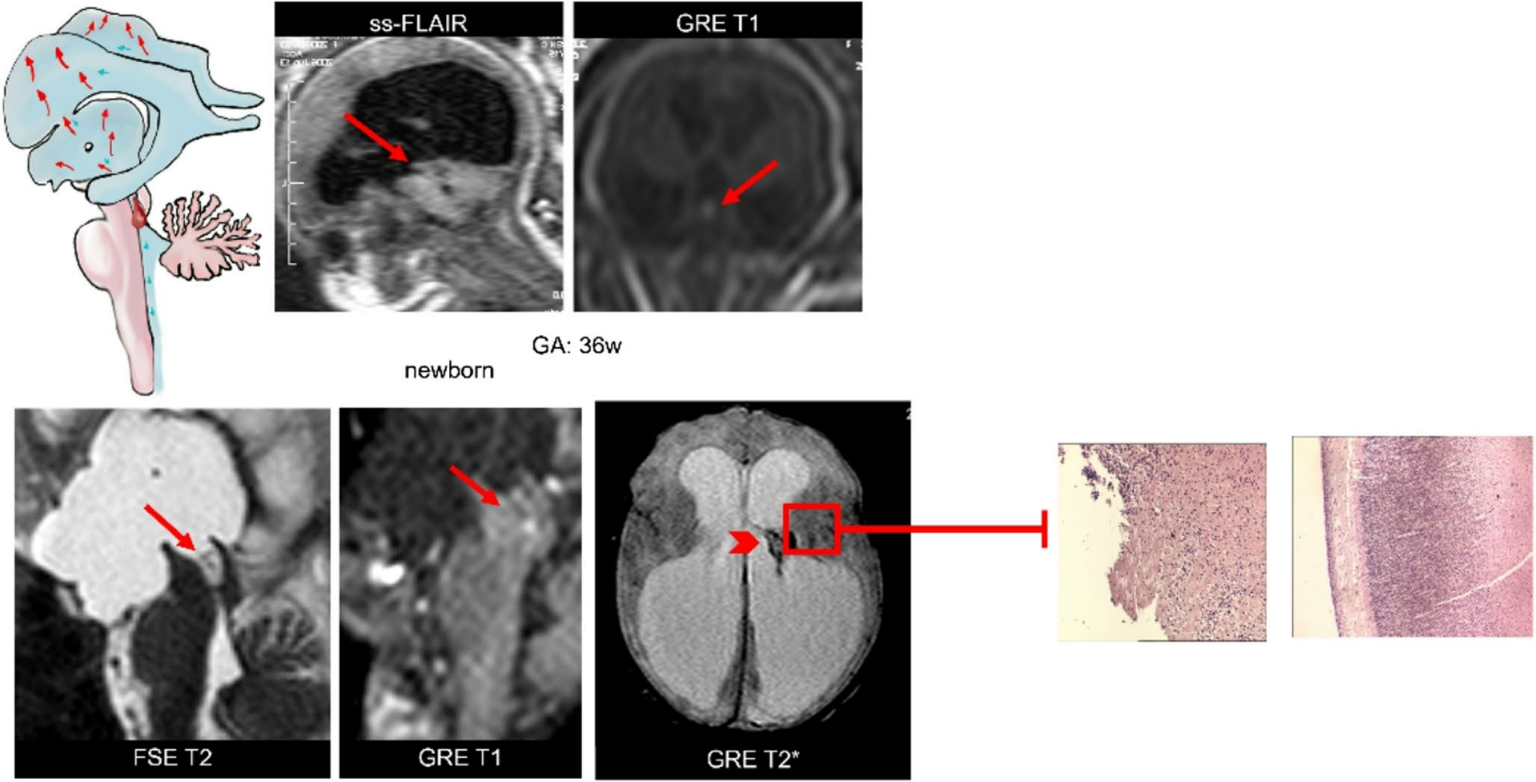
Post-hemorrhagic hydrocephalus: Sagittal ss-FLAIR and gradient echo coronal T1w MR images depict clot-debris obstructing third inferior aqueduct. Newborn MRI: Axial gradient echo T2*, fast spin-echo T2w and GRE-T1 images confirm the prenatal findings, including ependymal hemorrhage and necrosis (arrowhead on GRE-T2*). Microscopic anatomy shows ependymal fragmentation-debris and periventricular white matter rarefaction

Emerging evidence demonstrates the deleterious impact of intraventricular hemorrhage on prenatal neuronal staminal cells (NSCs) and neurogliogenesis (Fig. 9).

Studies of brain tissues from premature infants with intraventricular hemorrhage show disorganization of the neurogenic ventricular zone and suppression of NSC proliferation [46, 48, 49]. Investigations in animal models have begun to define the cellular and molecular mechanisms underlying the deleterious effects of hemorrhage on prenatal NSCs and cortical neurogenesis. Using a fetal mouse model of post-hemorrhagic hydrocephalus, Yung et al. reported that a blood-borne lipid (lysophosphatidic acid) causes aberrant activation of Rho/Rac signaling, leading to disruption of NSC attachment in the ventricular zone and the onset of fetal ventriculomegaly [50]. More recent investigations combining both fetal human brain tissue examinations and rabbit models of preterm birth have shown that intraventricular hemorrhage reduces Wnt signaling, resulting in NSC apoptosis and suppression of cortical neurogenesis [49, 51]. These findings highlight alterations in NSC signaling as disease mechanisms that initiate ventriculomegaly and cortical underdevelopment in post-hemorrhagic hydrocephalus. Surprisingly, pharmacological manipulations targeting the identified molecular alterations restored neurogenesis and prevented the development of hydrocephalus in the studied models [49, 50]. Such findings aim to improve the outcome of these patients, for example through the development of molecularly targeted drugs, and to modify prenatal counselling.

### Rhombencephalosynapsis

**Fig. 10:**
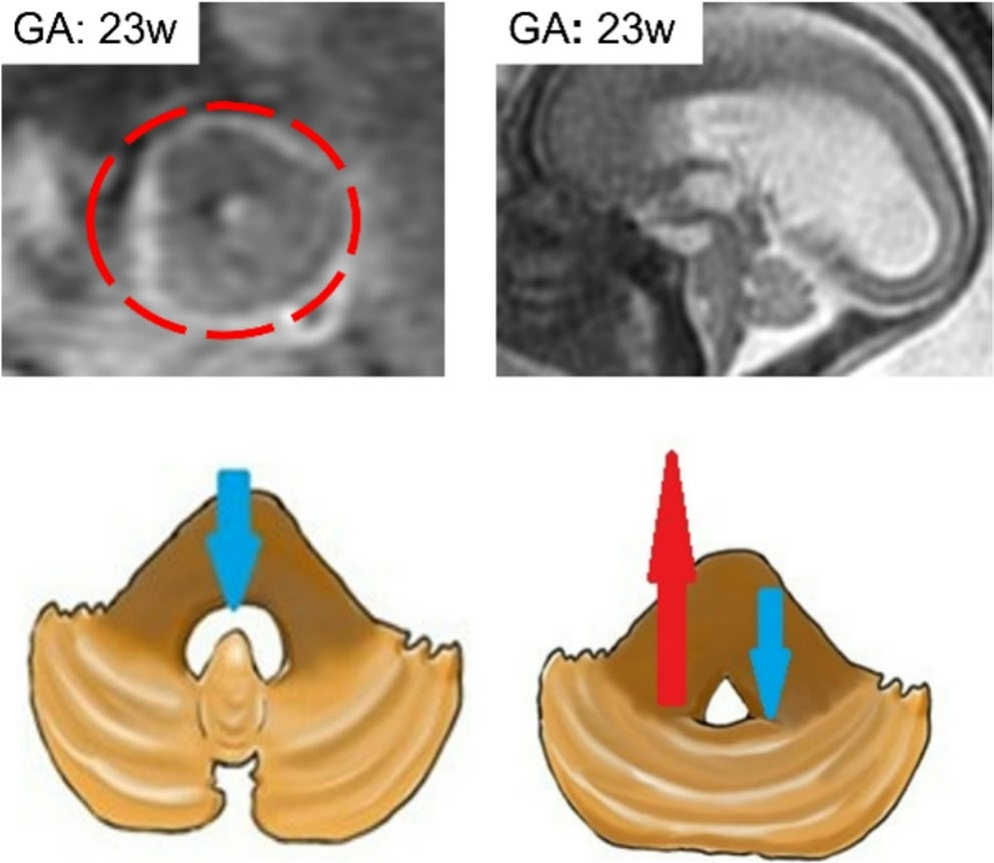
Axial and sagittal fast spin-echo T2w MR images of a 23-week fetus depict a typical agenesis of the vermis with fusion of the cerebellar hemispheres assuming a characteristic «keyhole» shape of fourth ventricle; this condition causes CSF reflux with hydrocephalus

Rhombencephalosynapsis (RES) is characterized by a deficient or absent vermis with hemispheres that are fused across the midline (Fig. 10). The cerebellar tonsils and deep cerebellar nuclei can also be fused. The severity of RES can be graded by the amount of remaining vermis. Milder forms of RES are often associated with normal or even large cerebellar size with both inferior and superior cerebellar ectopia. In contrast, severe and complete RES are often associated with cerebellar hypoplasia [52].

No evidence for extrinsic factors contributing to RES has been reported in the literature. Several studies have been reported chromosomal rearrangements, though no recurrent variants have emerged [53, 54, 55]. Partial RES was reported in child with a de novo mutation in the *CHAMP1* gene, but limited neuroimaging data were reported to substantiate this diagnosis [56]. In the absence of known genetic causes, several possible mechanisms for RES have been proposed based on the brain malformation and associated clinical features. For instance, one popular hypothesis is that RES results from a defect in dorsal-ventral axis formation [57, 58] similar to some forms of holoprosencephaly. In contrast, RES could be due to other abnormalities in specification of cerebellar vermis cell fates. Given the phenotypic overlap with VACTER-L, VACTERL-H (vertebral, anorectal, cardiac, tracheo-esophageal fistula, renal, limb, hydrocephalus) may share common mechanisms. One intriguing paradigm for birth defects caused by gene-environment interactions is vertebral defects resulting partial loss of Notch pathway function [59] because RES is associated with vertebral defects.

**Fig. 11:**
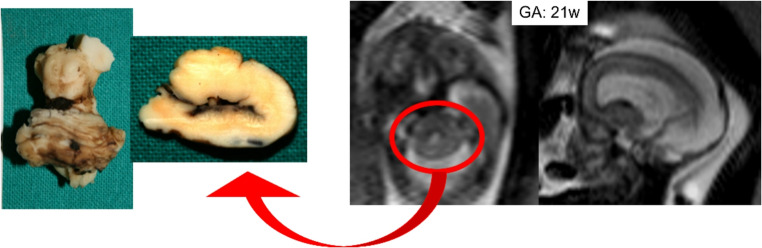
Axial and sagittal fast spin-echo T2w MR images of a 21-week fetus depict a typical agenesis of the vermis with fusion of the cerebellar hemispheres associated with severe hydrocephalus. These findings are well highlighted in gross anatomy specimen. Cerebellum shows the typical shape anomaly with flat and continuous wall from fusion of the hemispheres due to vermis absence

The presence of the following features on pre- or postnatal MR imaging should prompt close scrutiny for RES: aqueductal stenosis, substantial central cerebellar white matter on sagittal view, rounded fastigial point, absent posterior cerebellar notch (incisura), absent septum, and/or ventriculomegaly with a small cerebellum [60]. A large fetal autopsy series confirmed all these imaging findings, identified frequent Purkinje cell heterotopia, and found that the aqueductal obstruction was commonly due to atresia or forking, presumably a developmental rather than acquired abnormality [61] (Fig. 11).

Coexisting abnormalities have a strong impact on prognosis. Presence of more severe forms of holoprosencephaly is incompatible with life. Clinical presentation after birth ranges from mild truncal ataxia with normal cognition to severe cerebral palsy with epilepsy and mental retardation. Most patients have cognitive dysfunction, attention deficit and hyperactivity and motor milestones [62]. No prenatal or postnatal interventions have been described, though shunting may be required.

### Diencephalic-Mesencephalic junction dysplasia

**Fig. 12:**
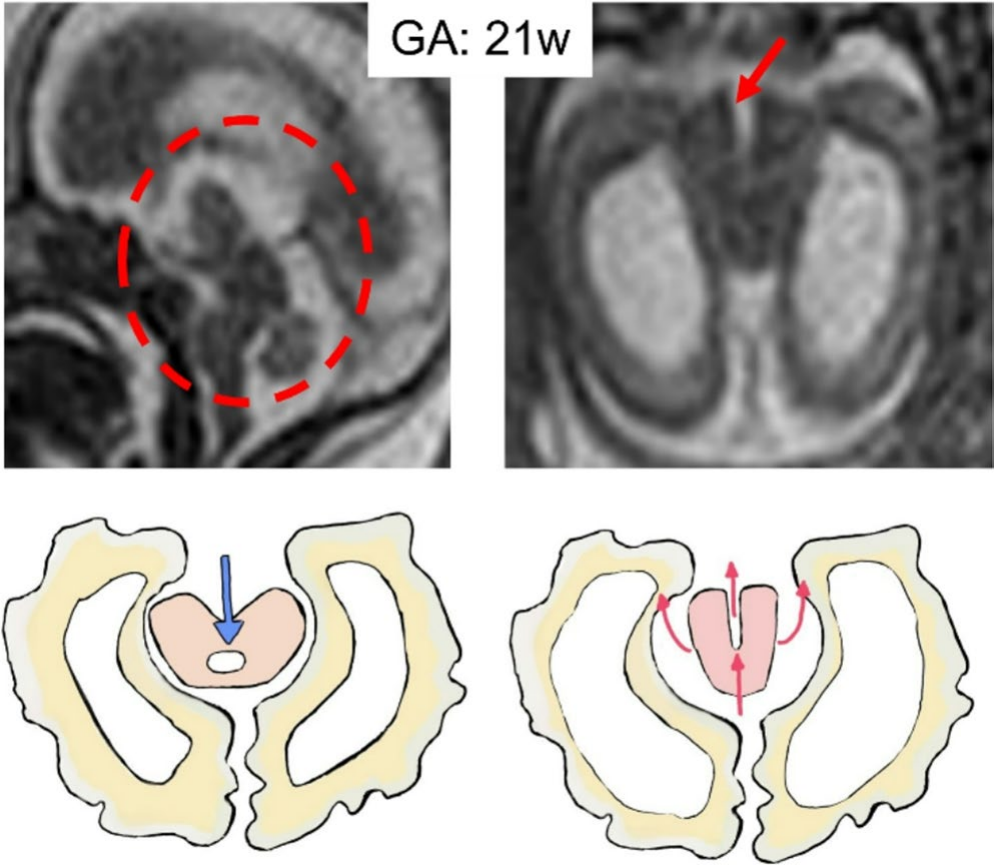
Sagittal and especially axial single-shot fast spin-echo T2w MR images of a 21-week fetus depict a deep longitudinal cleft in the ventral midline of midbrain contiguous with the third ventricle, a caudal displacement of diencephalic-mesencephalic junction and an abnormal butterfly-shaped midbrain shape; this condition may cause CSF reflux with hydrocephalus as showed in the illustration (normal versus pathologic CSF flow)

Diencephalic-mesencephalic junction dysplasia (DMJD) is described as an abnormal “butterfly like” midbrain configuration on axial MRI, with deep longitudinal cleft in the ventral midline of the midbrain contiguous with the third ventricle and caudal displacement of the DMJ. Associated hydrocephalus was present on fetal MRI in 91% of cases [63] (Fig. 12).

Without a clear etiology, prenatal genetic testing options in continuing pregnancies are limited. Recurrence risk is also unclear although autosomal recessive and X-linked inheritance patterns have been proposed [64]. Some studies identified bi-allelic pathogenic variants in the protocadherin‐12 gene (PCDH12) [65], L1CAM pathogenic/likely pathogenic variants and VRK1 bi-allelic pathogenic variants [66]– [67].

**Fig. 13:**
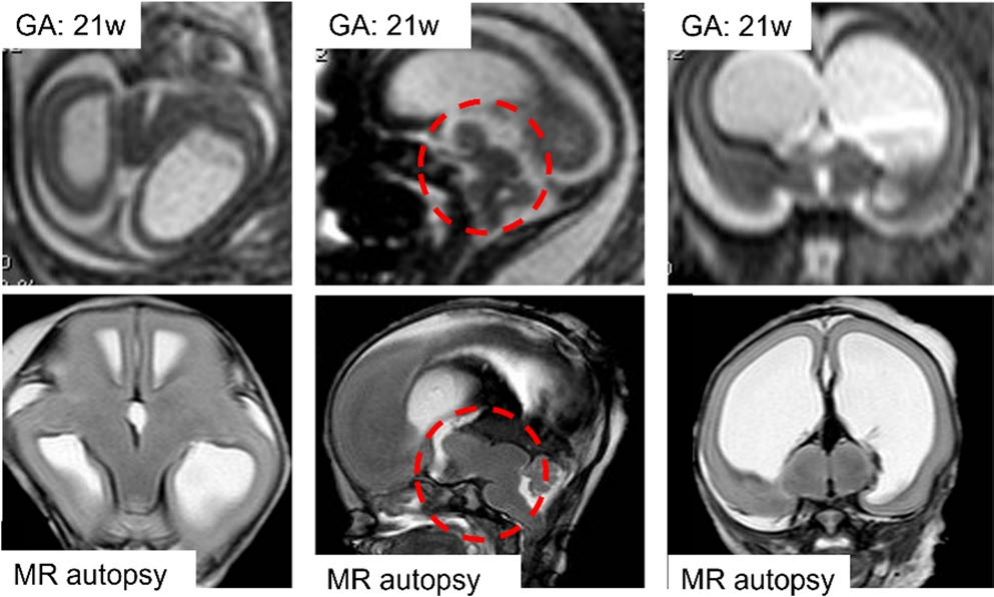
Axial, sagittal and coronal fast spin-echo T2w MR images of a 21-week fetus depict a poorly defined junction between the diencephalon and the mesencephalon, with a deep longitudinal cleft in the ventral midline of midbrain contiguous with the third ventricle, a caudal displacement of diencephalic-mesencephalic junction and an abnormal butterfly-shaped midbrain associated with hydrocephalus. These findings are confirmed on turbo spin-echo T2w MR-autopsy images

One pattern, designated DMJD Type A, was characterized by abnormal cleavage between the midbrain and hypothalamus in the axial plane, which included the “butterfly like” pattern [68] (Fig. 13). The second pattern (DMJD Type B) is characterized by abnormal cleavage between the midbrain and thalamus in the sagittal plane, with persistent parenchymal continuity between the midbrain and the mass intermedia of the thalamus [69]. Recently, a new patterning defect was identified with complete or near complete continuity among hypothalamus/thalamus/midbrain (DMJD Type C) [70]. The long-term clinical outcomes are related to both the primary structural differences and secondary manifestations of these brain anomalies, and both impact prognosis. In general, the clinical phenotype in Type A is the most severe, while DMJD Type B cases have been less severely neurologically impaired [69]. All cases with postnatal confirmation of DMJD Type C survived [70]. No treatment or intervention was described prenatally or postnatally, but treatments could be needed.

### Walker Warburg syndrome

**Fig. 14:**
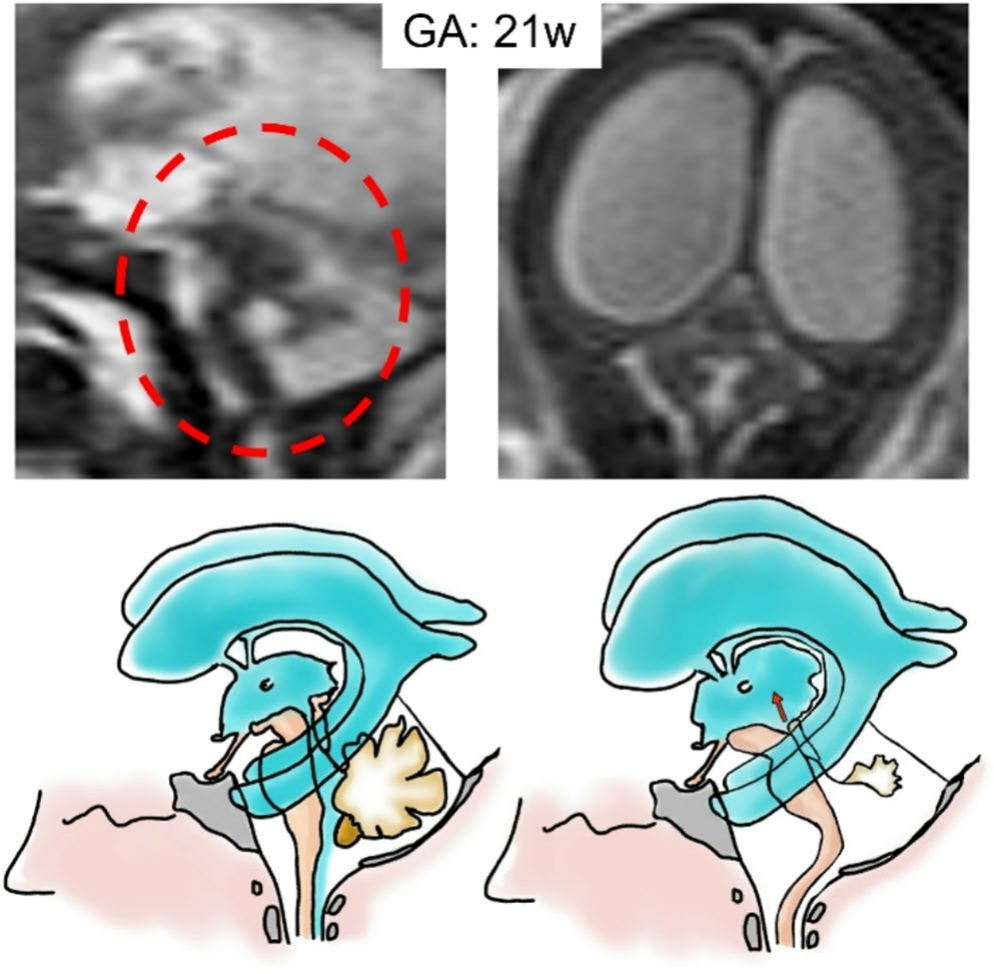
Sagittal and coronal single-shot fast spin-echo T2w MR images of a 21-week fetus stand for pontine hypoplasia, an enlarged quadrigeminal plate with fusion of the superior and inferior colliculi and a distinctive kinking in the dorsal pons; a second kinking may be present at the ventral cervical-medullary junction, forming the typical “z-shape” appearance of the brainstem. This malformation causes a CSF reflux in supratentorial sections with hydrocephalus (as showed by illustration in which normal and pathological brainstem morphology are depicted)

Walker-Warburg Syndrome (WWS) is a genetically heterogeneous disease presenting with congenital muscular dystrophy, type II lissencephaly, hydrocephalus, cerebellar malformations and eye abnormalities [71]. So far, only 10%–20% of cases can be confirmed by DNA analysis of mutations in the Protein O-Mannosyltransferase 1 (POMT1) gene [72]. DNA analysis in other genes besides POMT1 can be confirmative: fukutin [73]; FKRP [74] and POMT2 [75]. The overall incidence is unknown. Symptoms and signs are already present at birth and early infancy, and occasionally can be detected prenatally with imaging techniques. There may be a variety of anterior eye anomalies (cataracts, shallow anterior chamber, microcornea and microphthalmia, and lens defects) and a spectrum of posterior eye anomalies (retinal detachment or dysplasia, hypoplasia or atrophy of the optic nerve and macula and coloboma). Glaucoma or buphthalmos may be present. Brain abnormalities include migrational defect with type II lissencephaly (cobblestone type), hydrocephalus, vermal or general cerebellar hypoplasia and flat brainstem with small pyramids (Fig. 14).

The condition is usually lethal within the first few months of life, with almost all children dying by the age of three. Management is only supportive and preventive. If seizures develop, they usually need to be treated with anticonvulsants. A few children require a neurosurgical procedure such as shunting of hydrocephalus [76].

### Blake’s pouch cyst (Unperforated)

**Fig. 15:**
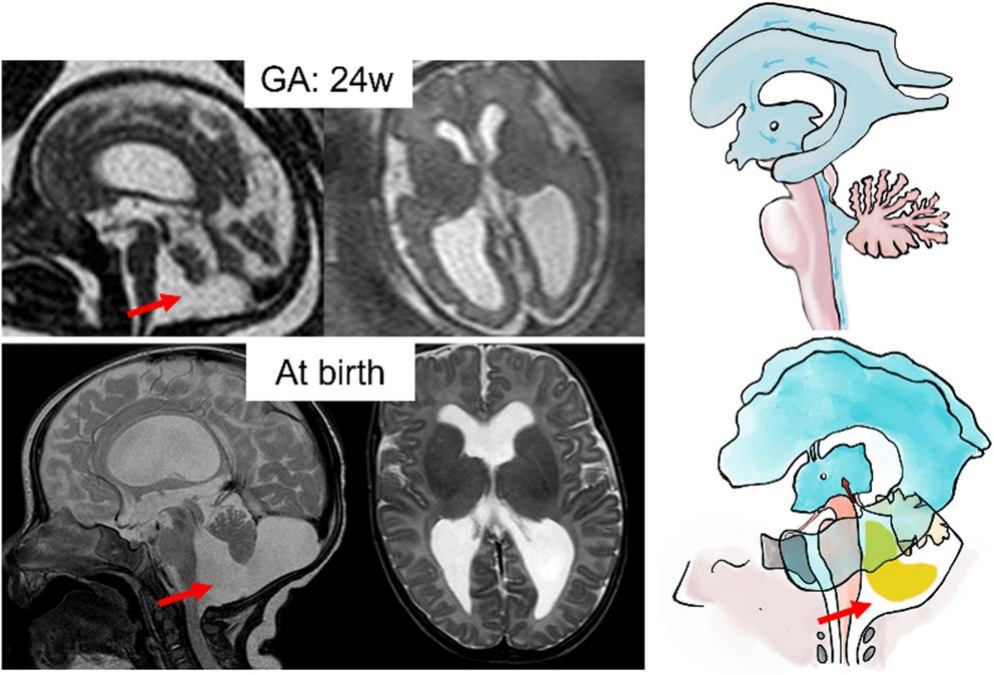
Sagittal and axial single-shot fast spin-echo T2w MR images of a 24-week fetus show obstructive hydrocephalus due to membranous obstruction of IV ventricle outlet associated with vermian rotation. These findings are confirmed at post-natal MRI. Illustration shows how an unperforated pouch, causing mass effect in posterior cranial fossa, induces a CSF reflux on supratentorial section compared to normal sample

In persistent (unperforated) Blake’s pouch, there is thought to be inadequate fenestration of both the foramen of Luschka, leading to imbalance of CSF egress into the subarachnoid space with consequent dilatation of the fourth ventricle. It has also been suggested by several cases in the literature that a sort of persistent Blake’s pouch phenotype can be “acquired” if the balance of CSF egress is upset by the presence of fetal intraventricular hemorrhage and fetal infection which result in tetraventricular dilatation and enlargement of the “cisterna magna” [77] (Fig. 15).

**Fig. 16:**
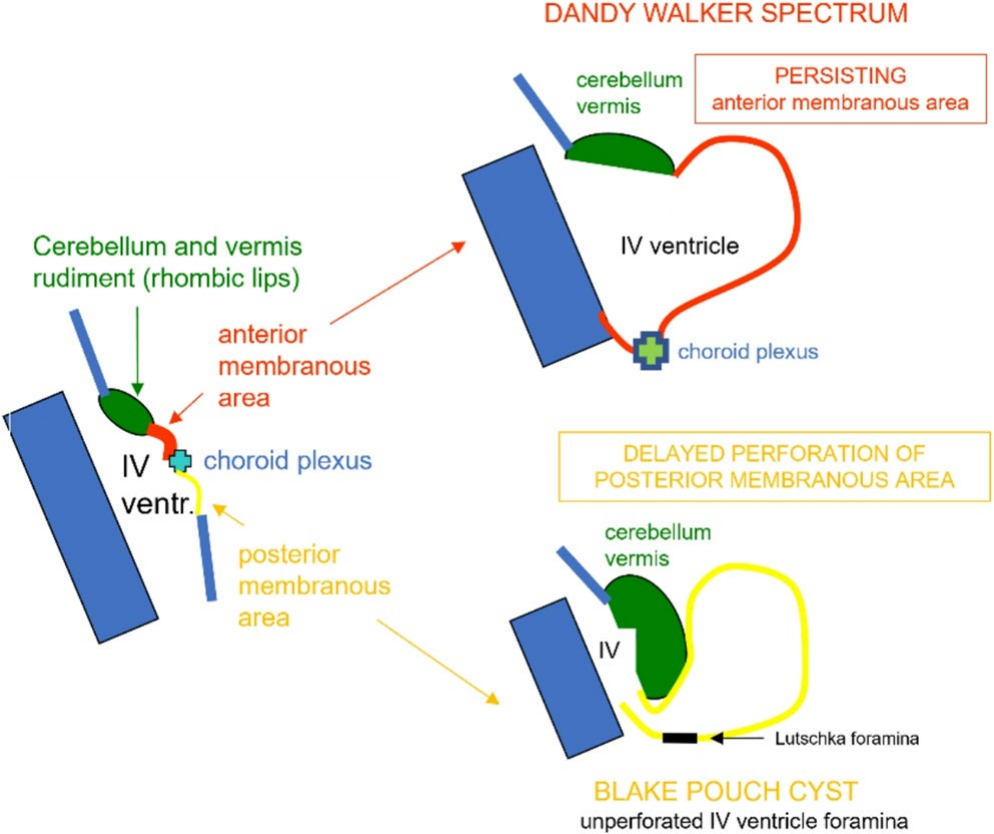
Illustration shows the pathogenic difference between Dandy Walker Spectrum (linked to anterior membranous area anomaly) and Blake’s pouch imperforation (linked to posterior membranous area anomaly)

The wall of Blake’s pouch can be depicted by ultrasound but more easily by MRI [78]. In terms of differential diagnosis, it appears essential to distinguish between the most common cystic malformations of the posterior cranial fossa [79] and with Dandy Walker malformation. In this regard, it is necessary to know, in addition to the differences in neuroimaging (Table 2), also the differences from an embryological point of view as shown in Fig. 16.

**Table 2:** Main neuroradiological differences between blake’s cyst and Dandy-Walker malformation

	Blake’s Cyst	Dandy-Walker Malformation
Cerebellar vermis	Intact and normal	Hypoplastic or absent
Fourth ventricle	Normal, but displaced posteriorly	Dilated and in pathological communication with an enlarged cisterna magna
Cisterna magna	Enlarged, but not associated with cerebellar malformations	Enlarged, with abnormal continuity between the fourth ventricle and cisterna magna
Atresia of the foramen of Magendie	Present, causing obstruction of CSF flow	Usually associated with abnormal communication
Associated findings	No cerebral or other malformation	Often associated with other CNS malformation

**Fig. 17:**
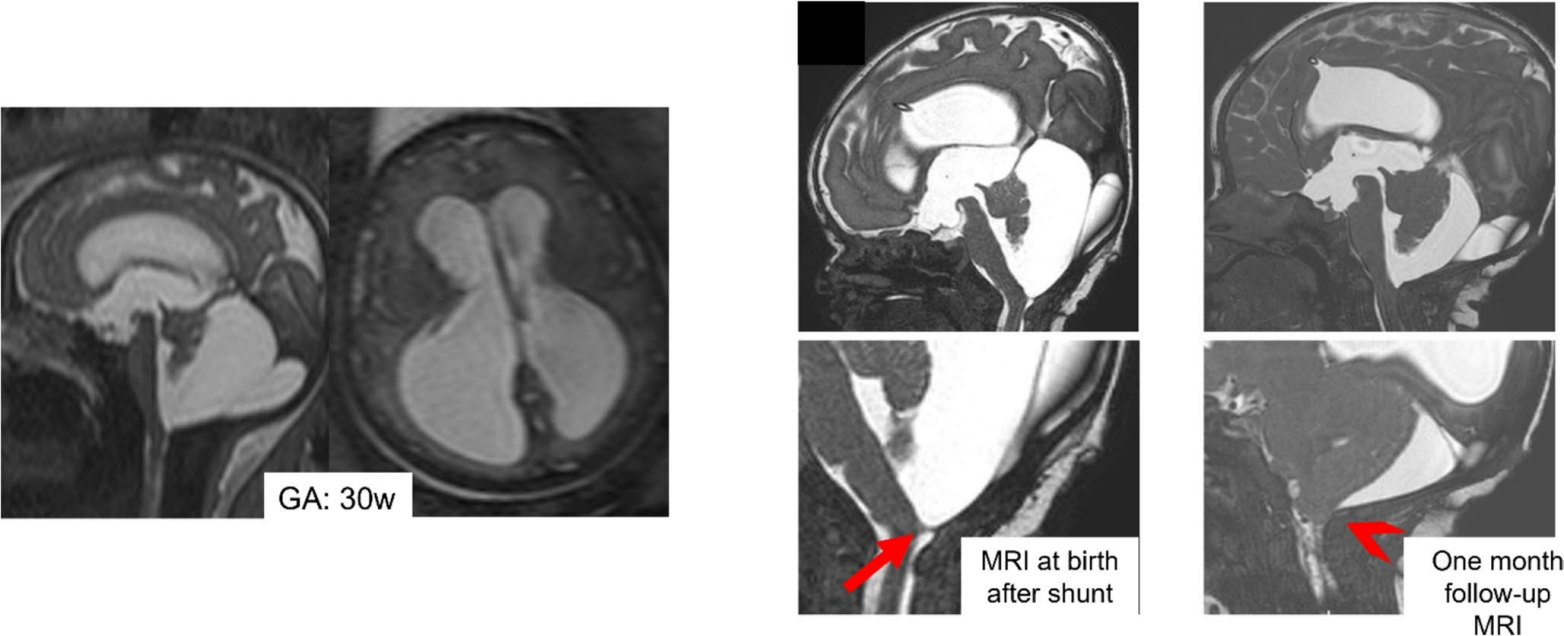
Sagittal and axial single-shot fast spin-echo T2w MR images of a 30-week fetus show obstructive hydrocephalus due to membranous obstruction of IV ventricle outlet. At birth, a shunt is placed with an almost complete return to the normal morphology of the PCF after one month

Isolated elevation/rotation of the vermis due to a persistent Blake’s pouch does not necessarily indicate an adverse outcome. Blake’s pouch cyst often regresses in-utero, after late perforation [80]. Expectant management is indicated, provided unusual vermian hypoplasia and other associated abnormalities are excluded [81]. In adults with Blake’s pouch cyst, after ventricular shunting, subtotal or total re-expansion of the cerebellar hemispheres and vermis can be seen as shown in Fig. 17.

#### Dural sinus malformation

**Fig. 18:**
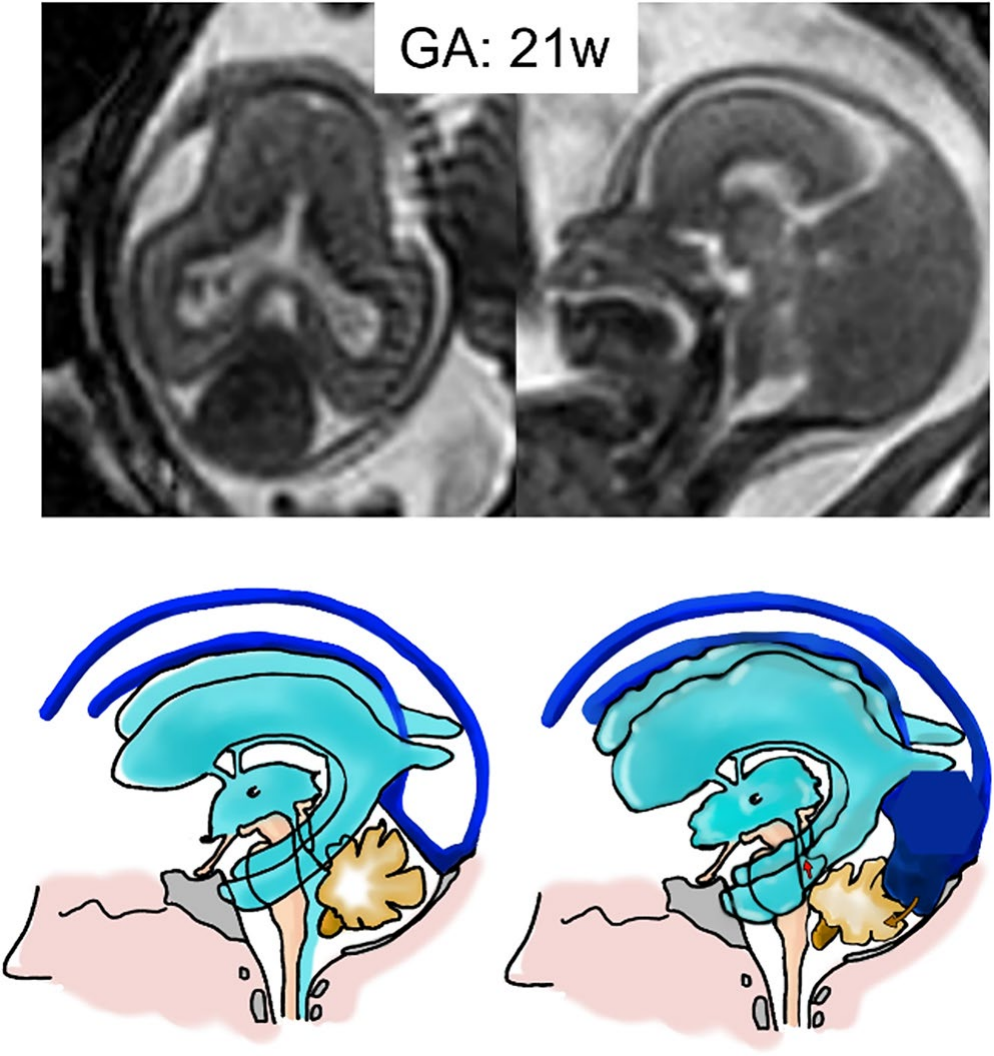
Sagittal and axial single-shot fast spin-echo T2w MR images of a 21-week fetus show well-defined triangular mass in the posterior fossa adjacent to the cerebellum; this mass due to dural sinuses enlargement compresses and anteriorly displaces the tentorial structures resulting in compression on the aqueduct and hydrocephalus. Illustration shows the differences between a normal case and a DSM one in the CSF flow dynamics

Dural Sinus Malformations (DSMs) are pathologic enlargements of dural venous sinuses and can be associated with arteriovenous shunts [82]. A DSM can precipitate thrombus formation [83]. DSM is an embryological abnormality resulting in a developmental defect. No etiologic correlation was found with thrombophilia or other hematologic disorders, TORCH infections, anemia, or pro-thrombotic risk factors [84]. Fetal DSMs are usually detected between 17- and 34-weeks during routine second or third trimester obstetric US screening. MRI is used next to clarify the diagnosis. DSM is shown as well-defined round or triangular masses in the posterior fossa adjacent to the cerebellum. Concomitant findings may include hydrocephalus, mass effect on adjacent cerebral parenchyma, and polyhydramnios [85] (Fig. 18).

In literature, no prenatal or postnatal treatments or interventions have been described; however, some studies suggest that it may be a proposed approach. Yang et al. [86] suggested endovascular embolization with enoxaparin sodium injections for thromboses and surgical interventions associated to ventricular shunt placement and anticonvulsant therapy for possible epilepsy. Sacco et al. [85] reported that a quarter of DSM pregnancies were terminated after diagnosis, and 10% of the remaining cases died perinatally. However, among the surviving cases, long-term follow-up indicated that the majority had a “normal” neurological outcome.

### Vein of Galen malformation

**Fig. 19:**
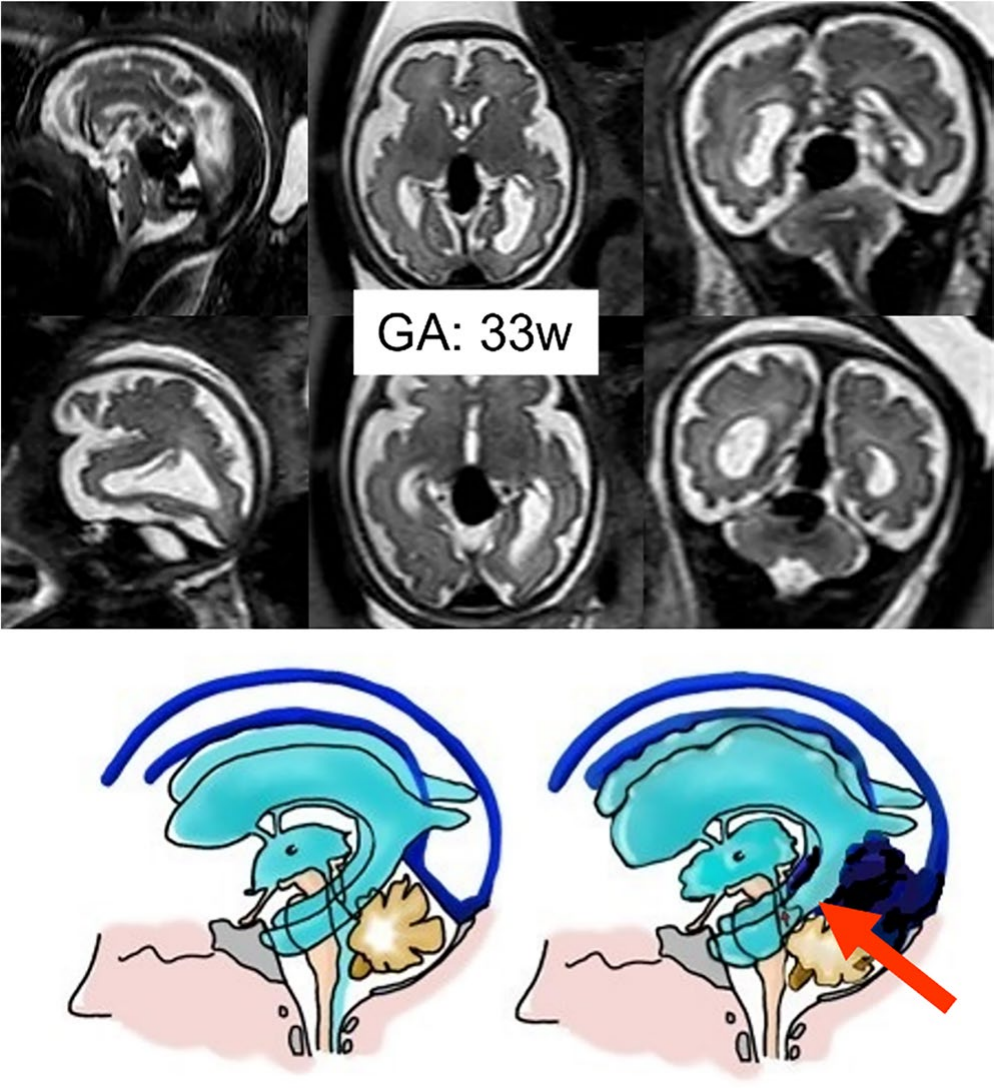
Sagittal, axial and coronal single-shot fast spin-echo T2w MR images of a 33-week fetus show well-defined ovoid mass due to aneurismatic dilatation of Galen’s vein associated with moderate hydrocephalus due mostly to aqueductal compression. Illustration shows the differences between a normal case and a VGM one in the CSF flow dynamics

Vein of Galen malformations (VGM) is the most frequent arteriovenous malformation in the fetus [87]. A VGM is hypothesized to occur when there is lack of involution of the prosencephalic vein because of either early occlusion or lack of formation of the straight sinus or due to continuous elevated blood flow through persistent abnormal communication of the choroidal arteries and the median pros encephalic vein [88]. MRI can identify flow void in pathological vessels and can verify the size of the VGM, which may demonstrate flow void or heterogeneous signal due to turbulence. Cerebral injury may manifest as ventriculomegaly and periventricular injury (Fig. 19).

Fetuses with VGM and cardiac failure, hydrops, or cerebral anomalies have a poor outcome. Without embolization, mortality in VGM is as high as 90%. With embolization: 10% demise; 10% severe disability; 16% moderate delay; and 74% neurologically normal [87].

Spontaneous thrombosis has been sometimes documented [89] however, early embolization may be often required.

### Antenatal surgery

Patient selection guidelines for intrauterine treatment of hydrocephalus were established by the International Fetal Surgery Registry: singleton pregnancy; absence of any other significant anomalies; progressive ventricular dilatation; presence of multispecialty team and consensus by the team to proceed; normal karyotype; gestational age less than 32 weeks or lung immaturity; adequate follow-up [90].

The main in-utero neurosurgical procedures are repeat cephalocentesis, endoscopic third ventriculostomy, and ventriculoamniotic shunt. Repeated cephalocentesis typically involves removing 20–120 mL of CSF under ultrasound guidance. The process stops if the fetal heart rate, monitored through continuous controls, slows. Ventriculoamniotic shunting is done percutaneously with a pigtail catheter under ultrasound supervision. The fetal lateral ventricle receives one end of the catheter, while the amniotic cavity receives the other. In third ventriculostomy, performed under fetal anesthesia, a tiny incision is created in the mother’s abdomen skin under ultrasound supervision, the fetal skull is pierced at the bregmatic fontanelle and the lateral ventricle is accessed. The foramen of Monro is located using a neuroendoscope and the third ventricle is penetrated. Newborns typically have ventriculoperitoneal shunting or endoscopic third ventriculostomy after ventriculoamniotic shunts are removed.

A comprehensive case study found that 67% of fetuses who had surgery and were monitored for more than three years had normal IQ (IQ above 70), 15% had mild or moderate handicap (IQ between 35 and 70), and 18% had severe handicap (IQ below 35). When hydrocephalus was discovered later (around the third trimester of pregnancy), the greatest outcomes were achieved [91].

In utero surgery for aneurysmal vein of Galen malformation (VGAM) is an innovative technique to treat this rare and serious cerebrovascular anomaly before birth. The malformation causes abnormal, high-velocity blood flow, overloading the fetal heart and leading to heart failure and brain damage. The procedure, performed using an ultrasound-guided fetoscopic approach, involves partial embolization of the malformed vessels to reduce the abnormal blood flow. Early studies indicate that this technique can significantly improve survival rates and reduce neurological complications compared to postnatal treatment [92, 93].

In utero surgery for VGAM is an experimental and highly complex procedure. The fetus is sedated. Using ultrasound guidance, the surgeon identifies the best entry point to reach the malformation. A thin cannula is inserted through the mother’s abdominal and uterine walls, reaching a fetal blood vessel, usually the umbilical artery or directly into an intracranial vessel. A microcatheter is advanced into the vein of Galen. An embolizing agent, such as cyanoacrylate glue or onyx particles, or metal coils is inserted to partially occlude the abnormal arteries and reduce blood flow in the vein of Galen. Fetal vital signs are monitored to assess cardiac and cerebral response. If the procedure is successful and the fetus tolerates the procedure, the catheter is removed and the vascular access is closed [94].

## Limitations

This study has several limitations. It is a retrospective, single-center analysis based on descriptive statistics, which limits the strength of causal inference. Protocols evolved between 2005 and 2024, potentially introducing technical heterogeneity. Inter-rater agreement was not assessed, and genetic confirmation was incomplete across the cohort. Perinatal and postnatal outcome data were available only for a subset of cases, reducing prognostic correlation. Finally, because this was conducted at a tertiary referral center, the results may not be fully generalizable to broader populations.

## Conclusion

Hydrocephalus includes a heterogeneous group of abnormalities that usually occur owing to derangements in the steps of brain embryology or as result of acquired insults. Neuroimaging plays a pivotal role in diagnosis, pre-surgical evaluation or generally in counseling. Correct imaging technique is critical in assessment of obstructive hydrocephalus. If it’s present, we have to determine the correct etiological diagnosis in order to give right information to clinicians and patients. Therefore, knowledge of brain embryology, hydrocephalus etiologies and the main imaging findings is essential for the radiologist, gynecologist and neurosurgeon. Here, we present an etiology-based educational framework that may assist radiologists and clinicians in structuring differential diagnosis and counseling. Its potential to inform management should be considered hypothesis-generating; prospective validation with standardized outcomes is required before it can guide interventions. Therefore, rigorous pre- and postnatal imaging follow-up and monitoring are essential.

## Data Availability

No datasets were generated or analysed during the current study.
